# Functional Analysis of the *ZmPR5* Gene Related to Resistance Against *Fusarium verticillioides* in Maize

**DOI:** 10.3390/plants14050737

**Published:** 2025-02-28

**Authors:** Wei Yang, Hongyu Cai, Yuanqi Zhang, Junheng Hao, Yaqi Ma, Qinxuan He, Kun Zhao, Honggang Li, Ayue Fang, Dalong Hou, Xuejiao Ren

**Affiliations:** 1 Agronomy Faculty, Jilin Agricultural University, Changchun 130118, China; weiy@jlau.edu.cn (W.Y.); cai384836624@163.com (H.C.); yuanqizhang2025@163.com (Y.Z.); 13704346808@163.com (J.H.); 18364926513@163.com (Y.M.); 19187956131@163.com (Q.H.); z1195471456@163.com (K.Z.); 18368366414@163.com (A.F.); 13488564131@163.com (D.H.); 2 Jilin City Academy of Agricultural Sciences, Jilin City 132102, China; 13604781196@163.com

**Keywords:** maize inbred line, J1259, *Fusarium verticillioides*, ear rot, *ZmPR5*, functional analysis

## Abstract

In this study, the gene *ZmPR5*, associated with resistance to ear rot, was identified through transcriptome data analysis of the maize inbred line J1259. The gene was subsequently cloned and its function was investigated. The *ZmPR5* gene comprises an open reading frame of 525 base pairs, encoding a protein of 175 amino acids. *ZmPR5* was overexpressed in Arabidopsis and the *ZmPR5EMS* mutant in maize, and they were subjected to q-PCR and measurements of antioxidant enzyme activities (POD, SOD, CAT, MDA), electrical conductivity, and chlorophyll content. The results indicate that the expression of *ZmPR5* is up-regulated upon infection by *Fusarium verticillioides*, with significant differences observed in the activities of POD, SOD, CAT, MDA, electrical conductivity, and chlorophyll content. The study found that *ZmPR5* is localized in the nucleus and interacts with *Zm00001d020492 (WRKY53)* and *Zm00001d042140 (glucA)*. Trypan blue staining revealed that the stained area in the *ZmPR5EMS* mutant of maize was significantly larger than that in B73. The expression of *ZmPR5* is closely associated with resistance to maize ear rot.

## 1. Introduction

Maize (*Zea mays*) is one of the most important staple crops worldwide. In recent years, the frequent occurrence of severe weather conditions has increasingly exacerbated the impact of diseases on maize yield [1]. Maize ear rot, primarily caused by fungal pathogens [2], leads to reduced production and diminished quality of maize [3]. It is reported that approximately 30 species of pathogens can cause maize ear rot, among which the most predominant are *Fusarium* species, such as *Fusarium verticillioides, Fusarium graminearum,* and *Fusarium oxysporum* [4]. Research has identified *Fusarium verticillioides* as a primary pathogen responsible for ear rot in maize. This fungus can infect maize through the silk, stem, and wounds on the ear, leading to the development of ear rot in pathogen-carrying seeds [5]. Additionally, *Fusarium verticillioides* is capable of producing a significant amount of mycotoxins, known as *Fusarium* mycotoxins, which include *trichothecenes*, *zearalenone (ZEA)*, and *fumonisins (FUM)*, among others. These toxins contribute to the occurrence of maize ear rot [6].

When plants are infected by pathogens, they often accumulate a class of defense proteins known as pathogenesis-related (PR) proteins [7]. These proteins can directly attack pathogens, disrupt the macromolecules of pathogen cell walls, and degrade pathogen toxins, playing a crucial role in plant defense against pathogen invasion [8]. Pathogenesis-related protein 5 (PR-5) belongs to the fifth family and exhibits various biological activities such as antifungal properties, β-1,3-glucanase activity, cold-resistant capabilities, and resistance to osmotic stress. PR-5 proteins are involved in the plant hypersensitive response (HR), signaling pathways, and systemic acquired resistance (SAR). Both biotic and abiotic stresses can trigger the response of PR proteins in plants [9].

Our laboratory obtained transcriptome data by infecting the ear rot-resistant maize inbred line J1259 and selected the candidate gene PR5, which is associated with maize ear rot, for further investigation. A comprehensive functional analysis was conducted through subcellular localization, yeast two-hybrid assays, phenotypic characterization of maize mutant plants, and phenotypic evaluation of Arabidopsis over-expression lines. This study aims to provide insights into the resistance mechanisms against maize ear rot and to guide the breeding of new maize cultivars with enhanced resistance to this disease.

## 2. Results

### 2.1. Bioinformatics Analysis Results

**Figure 1:**
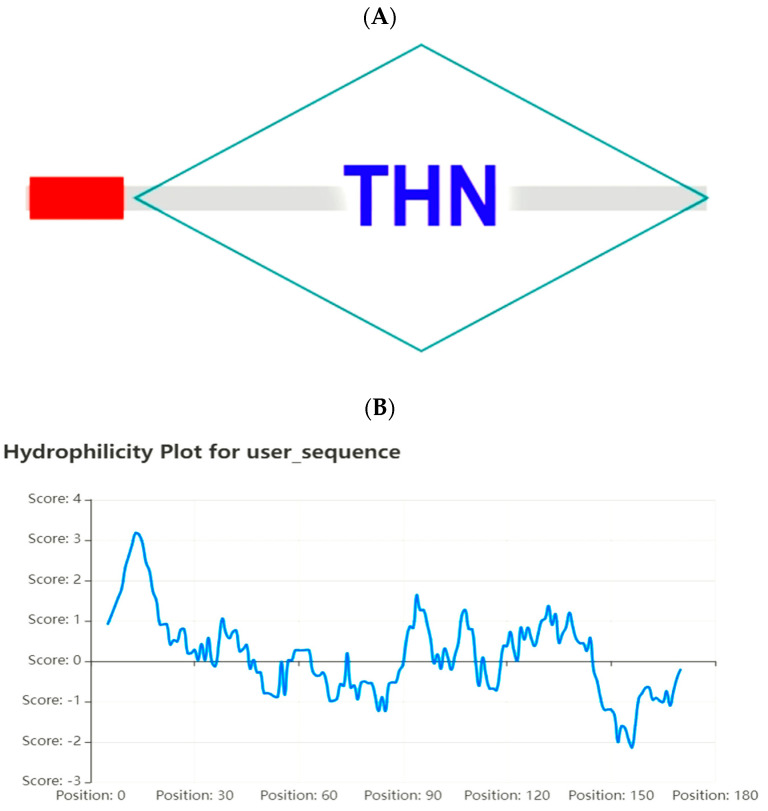
Hydrophobicity analysis of *ZmPR5* (**A**). The domain analysis of ZmPR5 revealed the presence of a THN domain (diamond block) and a signal peptide (red rectangle) (**B**).

The amino acid molecular weight of *ZmPR5* is 17,504.59 Da, with a molecular formula of C_755_H_1159_N_207_O_245_S_14_, comprising a total of 174 amino acids. The theoretical isoelectric point is 4.59. Based on the instability index of 41.01, it is inferred that the *ZmPR5* protein is unstable. Hydrophobicity analysis of the amino acids encoded by the *ZmPR5* gene is illustrated, where the horizontal axis represents the amino acid sequence number and the vertical axis represents hydrophobicity. A higher value indicates greater hydrophobicity, while negative values indicate hydrophilicity. Combined with the GRAVY (Grand Average of Hydropathy) value of 0.168, it is deduced that the *ZmPR5* protein is hydrophobic (Figure 1A).

An analysis of the conserved functional domains of the *ZmPR5* protein revealed that it contains a conserved THN domain and a signal peptide, suggesting it may localize to membrane systems, the extracellular space, or vacuoles. However, this study found that it localizes to the nucleus, suggesting the presence of a nuclear localization signal (NLS) (Figure 1B). *ZmPR5* belongs to the PR-5 family and is a thaumatin-like protein. Several members of this family have been shown to exhibit antifungal activity by inhibiting hyphal growth in vitro, potentially through a membrane permeabilization mechanism [10].

Through the subcellular localization prediction of *ZmPR5*, the probabilities were found to be nuclear 34.8%; cytoplasmic 21.7%; mitochondrial 21.7%; vesicles of the secretory system 8.7%; peroxisomal 4.3%; endoplasmic reticulum 4.3%; and vacuolar 4.3%. Therefore, the predicted result is nuclear, as this category has the highest proportion among all classifications.

**Figure 2:**
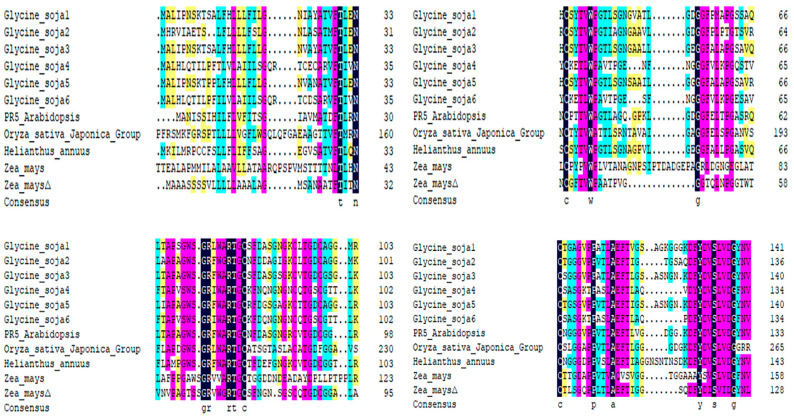
Analysis of sequence identity among *ZmPR5* homologous genes. Phylogenetic tree analysis of ZmPR5.

**Figure 3:**
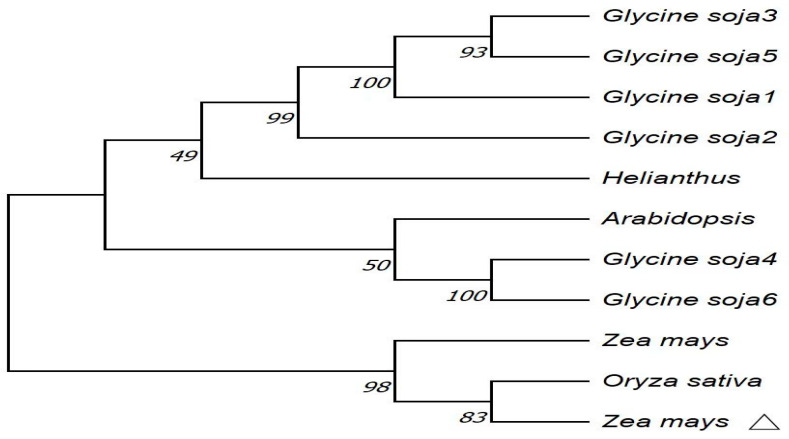
△ represents ZmPR5 in the phylogenetic tree analysis of ZmPR5. *Glycine soja*1:>NC_041015.1:c6520997-6508832, *Glycine soja*2:>NC_041005.1:c2731020-2729925, *Glycine soja*3:>NC_041018.1:40705826-40707557, *Glycine soja*4:>NC_041015.1:42491092-42493383, *Glycine soja*5:>NC_041015.1:c6524962-6523511, *Glycine soja6*:>NC_041014.1:19155679-19157544, *Arabidopsis thaliana*:>NC_003070.9:28177670-28179022, *Oryza sativa*:>NC_089039.1:c26859580-26858179, *Helianthus annuus*:>NC_035441.2:169632398-169633436, *Zea mays*:>NC_050099.1:c1779816-1778867.

In the NCBI database, homologous sequences of *ZmPR5* in other species were retrieved, and 10 sequences were obtained. The amino acid sequences of these species were compared with the *ZmPR5* protein sequence. Results showed that the *ZmPR5* protein possessed conserved domains almost identical to those of other PR proteins (Figure 2). In the phylogenetic tree analysis conducted using MEGA (version 10.2.1) software, dark colors represent highly conserved regions, while light colors represent regions with greater variability. The results indicate that the *ZmPR5* protein is relatively conserved and shows high similarity to rice PR-5 (LOC107280997) (Figure 3).

### 2.2. Subcellular Localization of ZmPR5

**Figure 4:**
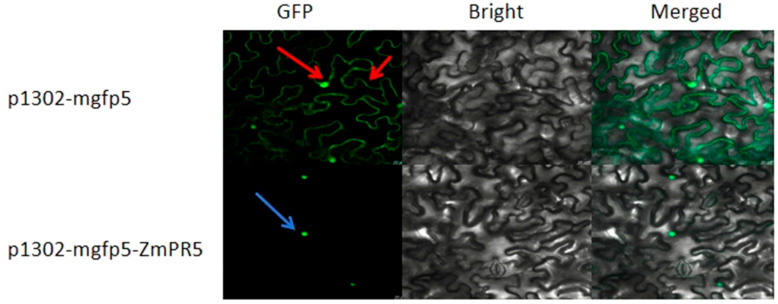
The p1302-mgfp5 vector exhibits green fluorescence in both the cell membrane and the nucleus (red arrow), whereas the p1302-mgfp5-ZmPR5 construct shows green fluorescence exclusively in the nucleus (blue arrow). Therefore, it is inferred that *ZmPR5* is localized in the nucleus.

The constructed p1302-mgfp5-ZmPR5 vector was introduced into *Agrobacterium* and injected into tobacco leaves for transient expression, with the empty p1302-mgfp5 vector serving as a control. GFP was fused to the C-terminus of the gene, and fluorescence localization was observed under a confocal microscope. The p1302-mgfp5 control showed expression in both the cell membrane and the nucleus, confirming successful infection, while the p1302-mgfp5-ZmPR5 construct exhibited green fluorescence specifically in the nucleus. The subcellular localization results indicate that *ZmPR5* is expressed in the nucleus of tobacco cells. These results are consistent with the predictions (Figure 4).

### 2.3. The Impact of Over-Expressing the ZmPR5 Gene in Arabidopsis

**Figure 5:**
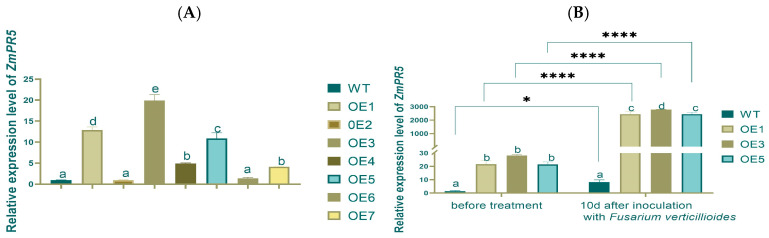
RELs of *ZmPR5* increased extremely significantly in Arabidopsis OEs after inoculation. (**A**) Selection of OE plants. (**B**) Before and 10 d after inoculation of T3 generation. Lowercase letters indicate significant differences between treatments at the *p* < 0.05 level.

Total RNA was extracted from seven T1-generation over-expression lines (OE1-OE7), and the Relative Expression Levels (RELs) of *ZmPR5* in each line were detected using quantitative fluorescence PCR(q-PCR). The results of the REL of *ZmPR5* in Arabidopsis showed that the OE3 group had significantly higher *ZmPR5* expression compared to the other groups. The OE1 and OE5 groups also exhibited relatively high expression levels, though lower than those of the OE3 group. Based on that, the OE1, OE3, and OE5 lines were selected for further research (Figure 5A).

**Figure 6:**
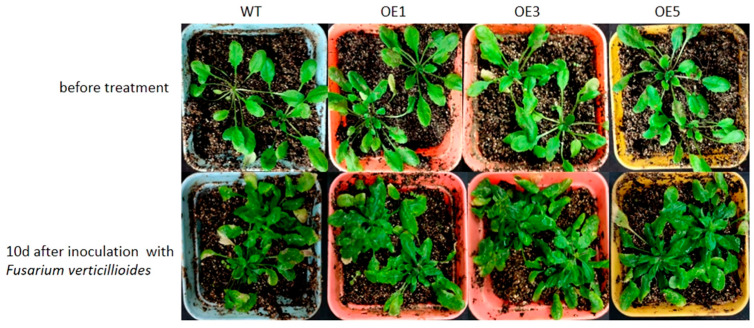
Before inoculation with *Fusarium verticillioides*, the leaves of Arabidopsis were green. Ten days after inoculation, wilting was observed in the leaves of wild-type (WT) Arabidopsis, and it was more severe compared to that in Arabidopsis OE plants.

The three selected lines were cultivated to the T3 generation and were sprayed with a spore suspension of *Fusarium verticillioides* when they reached the rosette stage. The RELs of *ZmPR5* and the activity of antioxidant enzymes were then measured [11]. Ten days after infection, both the wild-type and the OE Arabidopsis plants exhibited varying degrees of wilting (Figure 6). This study also found that *ZmPR5* is expressed in wild-type (WT) Arabidopsis, possibly due to the presence of homologous chromosomes of *ZmPR5* in Arabidopsis. By comparing WT and OE Arabidopsis before and after treatment, it is speculated that *ZmPR5* may enhance resistance to *Fusarium verticillioides*.

The RELs of *ZmPR5* in the WT group were the lowest before inoculation, while the RELs in the OE1, OE3, and OE5 groups were higher with significant differences. After inoculation, the WT group still exhibited the lowest REL of *ZmPR5*. The RELs in OE1, OE3, and OE5 showed significant differences compared to the WT group. Comparing pre- and post-inoculation, the RELs of *ZmPR5* increased by about 250 times on average, and significant and extremely significant differences were found in WT and OEs, respectively (Figure 5B).

As a common model organism, *Arabidopsis thaliana* is widely used for the study of gene function. SOD (superoxide dismutase) is a free radical scavenger in animals and plants, capable of eliminating superoxide radicals, reducing damage to the organism, delaying the aging process, and enhancing immunity. POD (peroxidase) is widely present in plants and is associated with respiration, photosynthesis, and the oxidation of auxins. POD activity is higher in aging tissues and weaker in young tissues. CAT (catalase) can catalyze the decomposition of hydrogen peroxide into oxygen and water, and it is found in all plant tissues, representing the plant’s antioxidant capacity and self-protection ability.

MDA (malondialdehyde) is the end product of membrane lipid peroxidation. Its accumulation can cause damage to membranes and cells, and an increase in MDA content indicates that the treatment may have induced oxidative stress, causing damage to cells.

Electrical conductivity reflects the ability of a substance to transmit electric current and can indicate the integrity and permeability of cell membranes. An increase in electrical conductivity suggests that the cell membrane may be damaged, leading to increased permeability.

Chlorophyll is a key pigment in photosynthesis. A low chlorophyll content reduces the efficiency of light absorption and energy transfer, thereby weakening the plant’s photosynthetic activity.

**Figure 7:**
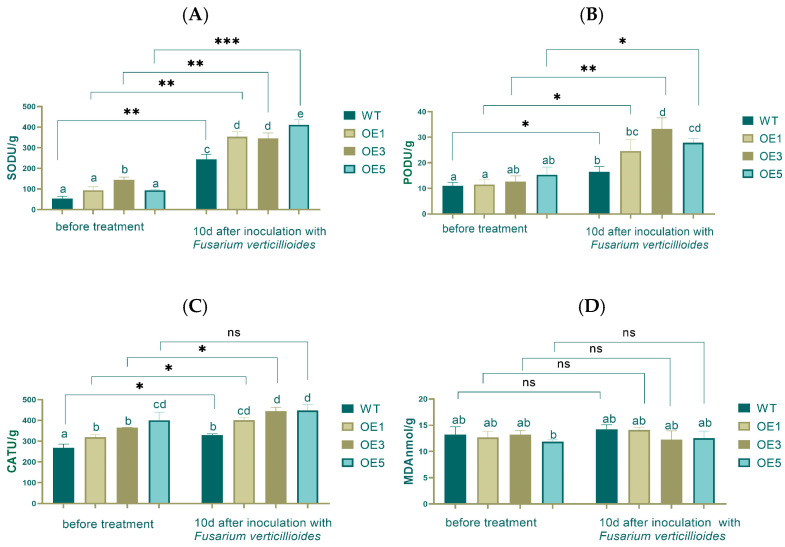
Over-expression of *ZmPR5* increases antioxidant enzyme activities and MDA content in transgenic Arabidopsis after inoculation (**A**–**D**). Lowercase letters indicate significant differences between treatments at the *p* < 0.05 level.

The measurement of antioxidant enzyme activity revealed that before inoculation, there was no significant difference in SOD activity between the WT, OE1, and OE5, but a significant difference with OE3. After inoculation, SOD activity increased 2.8 times on average, and a significant difference was found between WT and OEs. Before and after inoculation, very significant differences existed between WT, OE1, and OE3 and an extremely significant difference in OE5, respectively (Figure 7A).

There were no significant differences in POD activity between the over-expression lines and the WT. After inoculation, the POD activity increased, in particular by 3 times in OE3. Significant differences were found in WT, OE1, and OE5. A very significant difference was found in OE3 before and after inoculation (Figure 7B).

There were significant differences in CAT activity between WT and OEs before inoculation, and OE5 had the highest CAT activity. After inoculation, WT still had the lowest CAT activity, and significant differences were observed between the WT and OEs (Figure 7C).

Before the inoculation, there was no significant difference in MDA content between the WT group and the OE lines at the 0.05 level. After treatment, the MDA content generally increased, with the WT group showing the highest content, while the lines OE1, OE3, and OE5 had relatively lower MDA content with no significant differences among them. Comparing the pre- and post-treatment periods, there were no significant differences in MDA content in the WT, OE1, OE3, and OE5 groups after treatment (Figure 7D).

**Figure 8:**
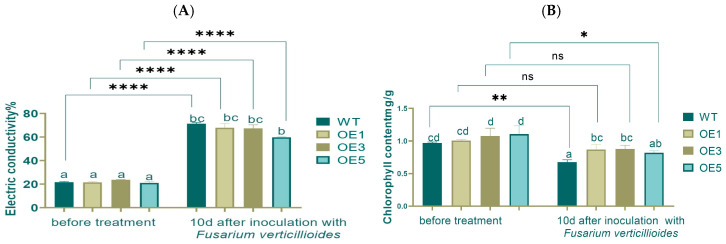
Electrical conductivity increased (**A**) and chlorophyll content decreased (**B**) 10 d after inoculation. Lowercase letters indicate significant differences between treatments at the *p* < 0.05 level.

Electrical conductivity can reflect the integrity and permeability of the cell membrane. Before or after inoculation, there were no significant differences between WT and OEs. While comparing pre- and post-inoculation, the electrical conductivity increased about 3 times, and the difference reached an extremely significant level (Figure 8A).

Low content of chlorophyll could weaken the plant’s photosynthetic activity. Before inoculation, the chlorophyll content showed no significant differences around 1.0 mg/g. After inoculation, the WT group showed the lowest levels of chlorophyll content at 0.7 mg/g, with OE1 and OE3 showing a significant difference compared with WT. Before and after inoculation, the chlorophyll content decreased, with very significant and significant differences in WT and OE5, respectively (Figure 8B).

### 2.4. ZmPR5 Interacts with WRKY53 and glucA

**Figure 9:**
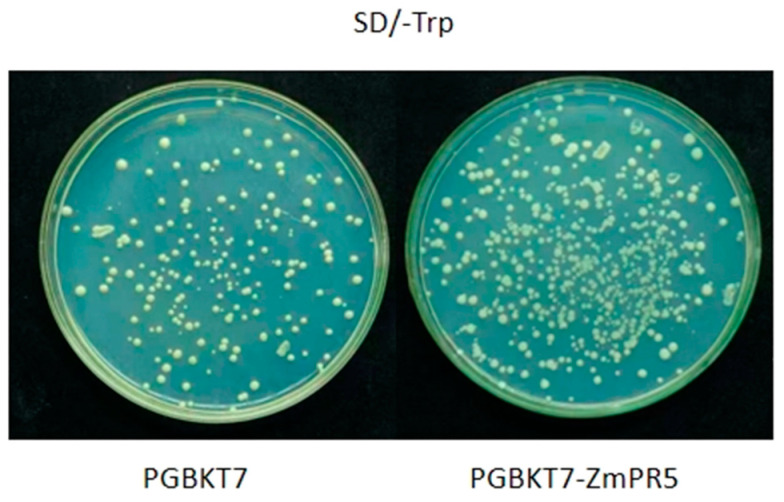
The consistent growth of PGBKT7-PR5 and PGBKT7 on SD/-Trp medium indicates that *ZmPR5* is non-toxic.

Constructed PGBKT7-ZmPR5 and PGBKT7 vectors were separately transformed into Y2HGold competent yeast cells and plated on SD/-Trp solid medium. Consistent growth was observed, indicating that *ZmPR5* does not exhibit toxicity (Figure 9).

**Figure 10:**
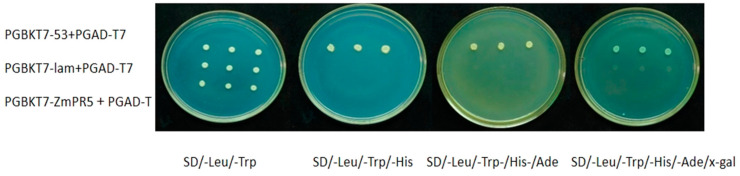
*ZmPR5* forms colonies on SD/-Leu/-Trp medium but does not form colonies on SD/-Leu/-Trp, SD/-Leu/-Trp/-His, SD/-Leu/-Trp/-His/-Ade, or SD/-Leu/-Trp/-His/-Ade/x-gal media, indicating that *ZmPR5* does not possess self-activation activity.

The PGBKT7-ZmPR5 and PGAD-T7 vectors were co-transformed and plated on SD/-Leu/-Trp solid medium. Colonies were then spotted onto SD/-Leu/-Trp, SD/-Leu/-Trp/-His, SD/-Leu/-Trp/-His/-Ade, and SD/-Leu/-Trp/-His/-Ade/x-gal solid media. It was observed that only the positive control (PGBKT7-53 + PGAD-T7) grew on SD/-Leu/-Trp/-His, SD/-Leu/-Trp/-His/-Ade, and SD/-Leu/-Trp/-His/-Ade/x-gal solid media, and turned blue on the SD/-Leu/-Trp/-His/-Ade/x-gal medium. In contrast, the negative control (PGBKT7-lam + PGAD-T7) and the experimental group only grew on the SD/-Leu/-Trp solid medium, indicating that *ZmPR5* does not possess self-activating activity (Figure 10).

In gene function research, protein–protein interaction experiments can verify whether the target protein interacts with other proteins of known function, thereby inferring its potential role or involvement in biological processes.

**Figure 11:**
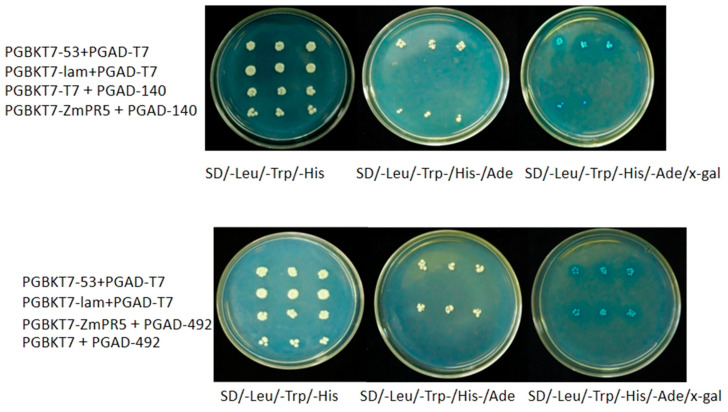
ZmPR5 interacts with Zm00001d020492 (WRKY53) and Zm00001d042140 (glucA).

Using the String database, potential interacting proteins of *ZmPR5* were predicted, revealing six genes that were predicted to interact with *ZmPR5*, *Zm00001d017276*, *Zm00001d003016*, *Zm00001d003015*, *Zm00001d017275*, *Zm00001d020492* (*WRKY53*), and *Zm00001d042140* (*glucA*). Results showed that *Zm00001d020492* (*WRKY53*) and *Zm00001d042140* (*glucA*) formed colonies on SD/-Leu/-Trp/-His/-Ade/x-gal solid medium, indicating that *ZmPR5* interacts with *Zm00001d020492* and *Zm00001d042140* (Figure 11).

### 2.5. The Impact of the Mutant ZmPR5 Gene in Maize

**Figure 12:**
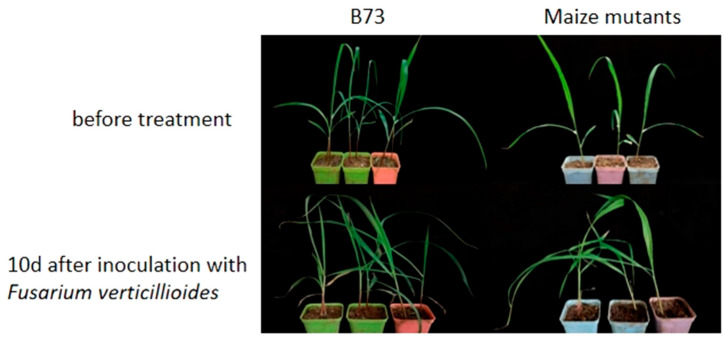
In maize mutants, lodging phenomena were observed 10 days after inoculation with *Fusarium verticillioides*.

EMS-mutant B73 lines, *zmpr5ems1* and *zmpr5ems2*, were selected based on significant differences in the RELs for *ZmPR5* compared to wild-type B73. A *Fusarium verticillioides* suspension was sprayed at the three-leaf stage, and the REL of *ZmPR5* and enzyme activity were measured. After 10 days of infection, both B73 and the mutants exhibited varying degrees of lodging (Figure 12).

**Figure 13:**
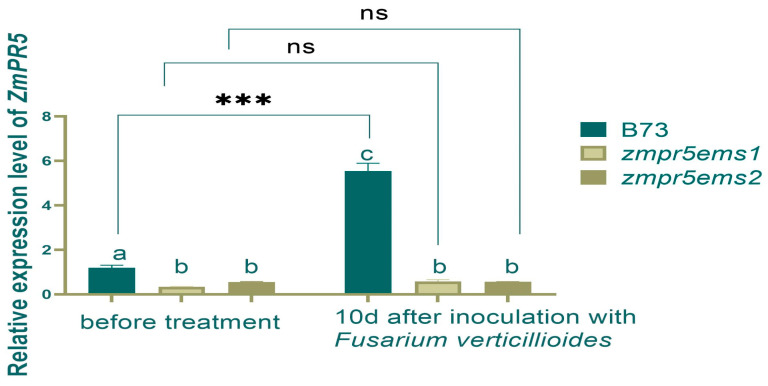
The REL of *ZmPR5* did not change significantly in maize mutants before and after inoculation in 10 d. Lowercase letters indicate significant differences between treatments at the *p* < 0.05 level.

Before inoculation, the RELs of *ZmPR5* in *zmpr5ems1* and *zmpr5em*s*2* were lower than in WT with significant differences. After inoculation, the REL of *ZmPR5* increased in WT by 2.5 times with an extremely significant difference. It showed a significant difference compared to *zmpr5ems1* and *zmpr5ems2.* No significant differences were observed before and after inoculation for REL in *zmpr5ems1* and *zmpr5ems2* (Figure 13).

**Figure 14:**
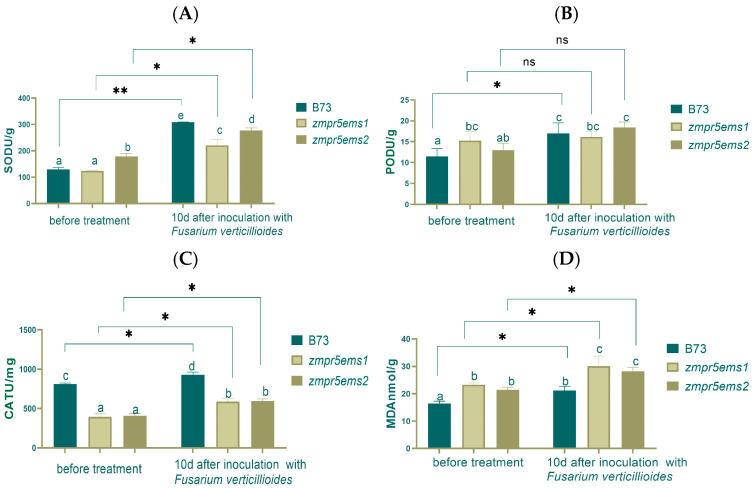
Antioxidant enzyme activities and MDA content increased significantly in maize B73 and mutants (**A**,**C**,**D**) after inoculation in 10 d. (**B**). The POD activity did not change significantly. Lowercase letters indicate significant differences between treatments at the *p* < 0.05 level.

This study found that before inoculation, the SOD activity in *zmpr5ems2* was higher than WT and *zmpr5ems1*, with a significant difference. After inoculation, SOD activity increased very significantly and significantly for WT and the mutants**,** respectively, with the WT exhibiting the highest SOD activity: 2.5 times higher than before inoculation (Figure 14A).

Before inoculation, the POD activity in B73 was the lowest, and there was a significant difference between WT and *zmpr5ems1*. After inoculation, no significant differences in POD activity were observed. POD activity rose with only WT showing a significant difference (Figure 14B).

The CAT activity in WT was the highest both before and after inoculation, with significant differences compared to that for *zmpr5ems1* and *zmpr5ems2*. The activity of CAT increased significantly (Figure 14C).

The MDA content bears a resemblance to the CAT activity for WT and OEs. After inoculation, the MDA content increased, and significant changes were observed (Figure 14D).

**Figure 15:**
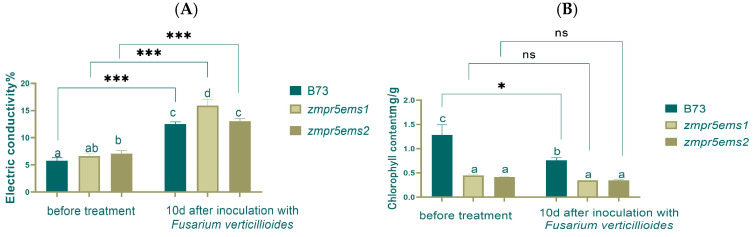
The electrical conductivity increased extremely significantly (**A**) and chlorophyll content did not change significantly (**B**) in maize 10 d after inoculation. Lowercase letters indicate significant differences between treatments at the *p* < 0.05 level.

This study found that electrical conductivity increased extremely significantly after inoculation by 2.5 times compared to before, with a significant difference between WT and *zmpr5ems1*. However, before inoculation, a significant difference existed only between WT and *zmpr5ems2* (Figure 15A).

Before inoculation, the chlorophyll content in WT was the highest, showing a significant difference with mutants. After inoculation, a significant difference still existed between WT and mutants, though chlorophyll content decreased significantly in WT (Figure 15B).

**Figure 16:**
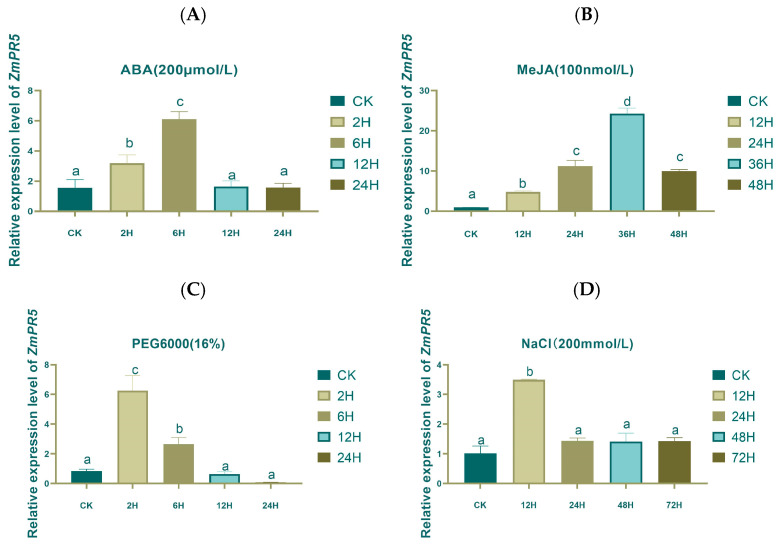
Expression pattern analysis of *ZmPR5*. (**A**) The REL of *ZmPR5* under ABA stress. (**B**) The REL of *ZmPR5* under MeJA stress. (**C**) The REL of *ZmPR5* under PEG6000 stress. (**D**) The REL of *ZmPR5* under NaCl stress. Lowercase letters indicate significant differences between treatments at the *p* < 0.05 level.

Analysis of maize expression patterns showed a pattern of increase and decrease for *ZmPR5* RELs, and the differences were significant. Under the ABA treatment, significant differences were observed at 2H and 6H (Figure 16A). MeJA resulted in significant differences observed at 12H, 24H, 36H, and 48H (Figure 16B). PEG6000 led to significant differences at 2H and 6H (Figure 16C). NaCl caused a significant difference observed at 12H. The results indicate that ABA, MeJA, PEG, and NaCl can all induce the expression of *ZmPR5* (Figure 16D).

### 2.6. Maize Mutant and Trypan Blue Staining

Trypan blue is a commonly used cell viability dye. Normally, viable cells possess intact cell membranes that can exclude trypan blue, preventing it from entering the cells. In contrast, cells that have lost viability or have compromised cell membranes exhibit increased permeability, allowing trypan blue to enter. Once inside, trypan blue binds nucleic acids (DNA and RNA), forming stable complexes that stain the cells blue.

**Figure 17:**
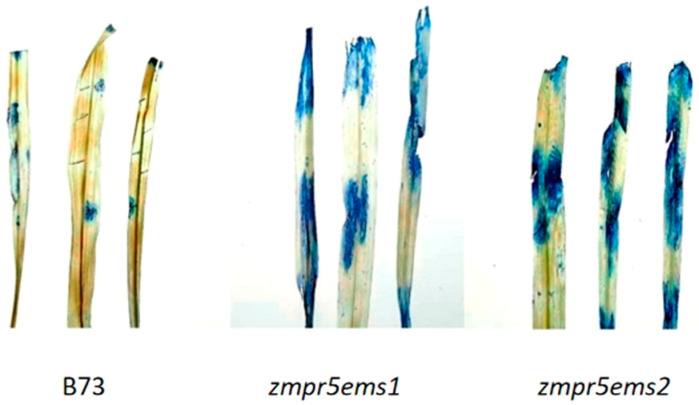
Stained maize leaves of *zmpr5ems1,2* indicated severe infection compared to B73 by employing trypan blue staining.

The results showed that the maize leaf was stained blue by trypan blue, indicating that the cell membranes in this region were damaged. By comparing the stained and unstained areas, it was evident that the mutant exhibits a higher degree of cell membrane damage than the wild-type maize B73, suggesting that the ZmPR5 mutant has lower resistance to *Fusarium verticillioides* compared to the wild type (Figure 17).

## 3. Discussion

Maize ear rot is primarily caused by *Fusarium verticillioides*. In the early stages, infected kernels exhibit white, pink, or reddish-brown mycelium [12]. As the pathogen spreads, the infected kernels typically develop pink or white streaks. The infection leads to the decay of maize kernels, and in severe cases, it can cause the maize ear to drop, thereby reducing both yield and quality [13].

PR (Pathogenesis-Related) proteins are a class of proteins that are significantly induced and expressed during pathogen infection [14]. PR proteins can be classified into 17 families, and most of these proteins can enhance plant resistance to pathogens [15]. PR-5 proteins are also known as thaumatin-like proteins (TLPs) [16]. TLPs exhibit a significant ability to inhibit the growth of pathogens [17].

PR-5 is an important family of plant defense proteins, including osmotin and osmotin-like proteins (OLPs) that possess various stress-resistant functions and can even degrade fungal cell membranes. Some members of the PR-5 family have been demonstrated to have antifungal capabilities [18] and activate defense responses [19]. The tobacco PR-5 protein, *NtPR5*, can strengthen the plant’s defense against further pathogen infections [20]. Soybean PR-5 is expressed in the roots and is involved in the plant’s defense against pathogens and its response to salt stress [21]. Phylogenetic tree analysis showed that *ZmPR5* exhibits the highest homology with rice PR-5. It is speculated that *ZmPR5* may improve resistance to *Fusarium verticillioides*.

The location of a protein within the cell determines its function. This study found that *ZmPR5* is subcellularly localized in the nucleus, which is a critical site for plant immune signal transduction and the regulation of gene expression [22]. *ZmPR5* may directly participate in regulating the expression of genes related to plant immunity. This contributes to the effective functioning of *ZmPR5* in plant immune responses.

Pathogens can induce an increase in reactive oxygen species (ROS) within plants. Antioxidant enzymes protect cells from oxidative damage by scavenging ROS and free radicals, maintaining intracellular redox balance, and enhancing plant resistance to stress and pathogens [23].

Research indicated that following *Fusarium verticillioides* infection, the REL of *ZmPR5* in Arabidopsis OE lines is 250 times that of the wild type (WT) on average, while in maize, the WT expression is 9.5 times that of the mutant. Studies revealed that the OE1, OE3, and OE5 lines exhibited stronger antioxidant enzyme activities compared to the wild-type Arabidopsis, with SOD and POD activities significantly increased post-treatment, showing notable differences from the wild type. This suggests that the over-expression of *ZmPR5* can enhance the antioxidant capacity of Arabidopsis. Furthermore, the CAT content also rose after treatment, further confirming the significant role of ZmPR5 in the antioxidant enzyme system. However, the MDA content in the OE lines is relatively lower post-treatment, with no significant difference compared to the wild type, possibly because the over-expression of the *ZmPR5* gene somewhat mitigates the process of cell membrane lipid peroxidation, aiding in the defense against oxidative stress caused by pathogen infection. The changes in antioxidant enzyme activities in the mutants after *Fusarium verticillioides* infection also significantly differ from those in B73, with SOD and CAT activities significantly lower than in B73 post-treatment. Meanwhile, the difference in POD activity is not significant, possibly due to the different mechanisms of action of various antioxidant enzymes in the disease resistance process. The changes in MDA content further confirm the more severe cell membrane damage in the mutant, with MDA content significantly higher than in B73 post-treatment.

Studies on electrical conductivity and chlorophyll content show an increase in electrical conductivity and a decrease in chlorophyll content, while the chlorophyll content in over-expressed Arabidopsis is significantly higher than in the WT, and in maize, that in the WT is significantly higher than in the mutant. Pathogen invasion can cause cell membrane damage, leading to increased electrical conductivity, with the lack of difference possibly due to the early stage of infection or the complexity of pathogen–plant interactions [24]. Pathogens cause the accumulation of ROS in plants, which attack chloroplast molecules, leading to chlorophyll degradation and reduced chlorophyll content [25].

Trypan blue staining studies show that the stained areas in WT leaves are significantly smaller than in the mutant, indicating less cell membrane damage in the WT during *Fusarium verticillioides* infection.

Through a yeast two-hybrid assay, it was discovered that two genes, *Zm00001d020492* (*WRKY53*) and *Zm00001d042140* (*glucA*), interact with *ZmPR5. WRKY53* is a transcription factor involved in regulating plant disease resistance responses [26]. The interaction with *ZmPR5* may suggest that *ZmPR5* plays a significant role in regulating the expression of disease resistance genes. *GhWRKY53* regulates cotton resistance to Verticillium wilt through the JA and SA signaling pathways [27]. The *WRKY53* transcription factor enhances disease resistance in Chinese wild grape by interacting with *MYB14* and *MYB15* [28].

Glutamate is a multifunctional metabolite and signaling molecule that is associated with plant growth, development, and defense responses [29]. It is hypothesized that *ZmPR5* may establish a certain connection between sugar metabolism and disease resistance responses in plants [30]. Through the analysis of cis-acting elements in *WRKY53* and *glucA*, it was discovered that these genes possess cis-acting elements responsive to ABA, MeJA, and IAA, which may potentially activate gene expression through these regulatory elements [31]. However, whether this gene confers resistance to *Fusarium verticillioides* requires further validation. The discovery of these interacting proteins provides new clues for in-depth research into the functional mechanisms of the *ZmPR5* gene, contributing to the elucidation of its specific role in the plant disease resistance process.

Analysis of expression patterns reveals that under ABA treatment, the expression of *ZmPR5* increased at 2H and 6H, with significant differences, indicating that *ZmPR5* may play an important role in the ABA signaling pathway and participate in regulating plant responses to drought and other stresses. Under MeJA treatment, the expression of *ZmPR5* rose at 12H, 24H, 36H, and 48H, showing significant differences, suggesting that *ZmPR5* had an important role in plant defense responses and may be involved in regulating plant resistance to biotic stresses such as pathogens. PEG6000 treatment, which simulates drought stress, results in increased expression of *ZmPR5* at 2H and 6H, with significant differences, further confirming its function in plant drought resistance. Under NaCl treatment, the expression of *ZmPR5* increased at 12H, with significant differences, indicating that *ZmPR5* also played a role in plant salt tolerance. Those results demonstrated that the *ZmPR5* gene can be induced to become expressed under various stress conditions, exhibiting broad stress resistance functions.

In summary, over-expression of the *ZmPR5* gene can enhance the activity of antioxidant enzymes, improve the stability of cell membranes, protect photosynthesis-related pigments, and thereby enhance the disease resistance capabilities of plants.

## 4. Materials and Methods

### 4.1. Experimental Maize Materials and Pathogenic Fungi

In the preliminary screening, ROS enzyme activity of the plants derived from the progeny of B73 (WT) were analyzed, which exhibits resistance to ear rot and was selected and preserved by the Corn Breeding Innovation Team at Jilin Agricultural University. The *Fusarium verticillioides* used in this study was provided by the same team and isolated from ear rot samples collected by the team. It was purified through single-cell culture and verified using Koch’s postulates.

The activated *Fusarium verticillioides* was inoculated onto PDA medium and prepared into a spore suspension with a concentration of 5 × 10^6^ spores/mL for subsequent use.

### 4.2. RNA Extraction and ZmPR5 Gene Cloning

RNA was extracted from successfully infected kernels using the TRIzol reagent kit. The quality of the RNA was verified to meet standards, followed by reverse transcription and q-PCR detection of the candidate gene. Specific primers were designed, and the amplification products were separated on a 1.2% agarose gel and extracted. These products were then cloned into the pMD18-T vector for sequencing, and vectors with correct sequencing results were selected.

### 4.3. Quantitative Analysis and Sequence Analysis of ZmPR5

Primers were designed based on the cloned *ZmPR5* sequence (*Zm00001d03115*). Quantitative detection was performed on maize and Arabidopsis treated with *Fusarium verticillioides*. At 0, 48 and 96 h post-inoculation, the mRNA expression levels of PR5 were detected using real-time q-PCR. β-actin was used as a control. Real-time RT-PCR amplification was conducted using SYBR mix (Shanghai China), and the RELs of the target gene were calculated using the 2^−ΔΔCt^ method [32].

The amino acids encoded by the *ZmPR5* gene were analyzed using the ExPASy-Protparam online tool (https://web.expasy.org/protparam, accessed on 23 February 2025). The hydrophobicity of the protein was analyzed using the Novopro online tool (https://www.novoprolabs.com, accessed on 23 February 2025). The subcellular localization of *ZmPR5* was predicted using Plant-mPLoc (http://www.csbio.sjtu.edu.cn/bioinf/plant-multi/, accessed on 23 February 2025), and the structural domains of *ZmPR5* were analyzed using Interpro (https://www.ebi.ac.uk/interpro, accessed on 23 February 2025). We performed an analysis of homologous genes in the *ZmPR5* family using DNAMAN (https://www.dnaman.net/, accessed on 23 February 2025) and MEGA (version 10.2.1).

### 4.4. Subcellular Localization Results of ZmPR5

Primers containing *Bgl II* and *Spe I* restriction sites were designed to insert the target gene *ZmPR5* into the p1302-mgfp5 vector. The constructed p1302-mgfp5-ZmPR5 vector and the empty p1302-mgfp5 vector were separately transformed into *Agrobacterium* GV3101. MES (2-morpholinoethanesulfonic acid) and AS (acetosyringone) were added to the bacterial culture [33]. When the OD600 of the bacterial culture reached 0.8, it was centrifuged and resuspended using a resuspension solution. After standing for approximately 3 h, the prepared *Agrobacterium* was injected into *Nicotiana benthamiana.* After 2 days of cultivation, the samples were observed and photographed under a Leica confocal microscope.

### 4.5. Over-Expression of ZmPR5 in Arabidopsis

Primers containing *Bgl II* and *Pac I* restriction sites were designed to insert the target gene *ZmPR5* into the pCambia3301-4MYC vector. The constructed vector was then transformed into *Agrobacterium* GV3101. Using the *Agrobacterium*-mediated floral dip method [34], Arabidopsis at the bolting stage was infected to obtain over-expressed Arabidopsis lines. Transgenic Arabidopsis T2 seeds were harvested from lines with expression levels significantly different from those of the wild type (WT). These seeds were cultivated to the T3 generation, and when the plants reached the rosette stage, they were sprayed with a *Fusarium verticillioides* spore suspension. The expression levels of *ZmPR5* and the enzyme activity in Arabidopsis were subsequently measured by using the T3 generation as duplications for each over-expression type.

### 4.6. ZmPR5 Yeast Two-Hybrid Assay

Primers containing *BamH I* and *EcoR I* restriction sites were designed to insert the target gene *ZmPR5* into the pGBKT7 vector. The constructed plasmid and the control pGBKT7 plasmid were separately transformed into Y2HGold competent yeast cells and plated on SD/-Trp solid medium. The growth of the colonies was observed to determine whether PR5 exhibited toxicity. The pGBKT7-ZmPR5 and pGADT7 plasmids were co-transformed into Y2HGold competent yeast cells and plated on SD/-Leu/-Trp solid medium. Colonies were spotted onto SD/-Leu/-Trp, SD/-Leu/-Trp/-His, SD/-Leu/-Trp/-His/-Ade, and SD/-Leu/-Trp/-His/-Ade/X-gal solid media for autoactivation verification. The interacting proteins of *ZmPR5* were predicted using String and subsequently validated.

### 4.7. Phenotypic Analysis of ZmPR5 Mutant Expression Pattern Analysis, and Trypan Blue Staining

The maize mutant *zmpr5ems* (purchased from the EMS mutant library http://maizeems.qlnu.edu.cn, accessed on 20 November 2023) was derived from the inbred homozygous maize line B73 advanced to the T3 generation and sown alongside the inbred maize line B73 in nutrient soil. At the three-leaf stage, mutant plants were sprayed with a *Fusarium verticillioides* spore suspension, and the expression levels and enzyme activities of the treated maize were subsequently measured. Leaves were collected from the maize, and small incisions were made on the leaves using a scalpel. A 1 cm × 1 cm agar block containing *Fusarium verticillioides* was placed upside down on the wound. After two days of infection, the leaves were stained with trypan blue staining solution for one day. The leaves were then rinsed once with 95% ethanol, followed by boiling in 95% ethanol for 5 min. The ethanol was replaced, and the boiling process was repeated until complete decolorization was achieved. The samples were then observed and photographed [35].

### 4.8. Enzyme Activity Electrical Conductivity and Chlorophyll Measurement

SOD activity was measured using the nitroblue tetrazolium (NBT) photoreduction method [36]. CAT activity was determined using the ultraviolet absorption method [36]. POD activity was assessed using the guaiacol method [37]. MDA content was measured using the TCA method [38]. Electrical conductivity was measured using a DDS-307 conductivity meter (Leici, Shanghai, China), and the relative electrical conductivity was calculated [39]. Chlorophyll content was determined following the measurement method described by Xu Fenfen [40].

### 4.9. Data Processing

Data analysis was performed using Excel 2019. The LSD method in SPSS 17.0 was used for significance analysis (*p* < 0.05). Graphs were created using GraphPad Prism 9.5 software. We repeated the data analysis three times. The significance levels for each statistical test are denoted as follows: ns: not significant 0.01 < *p* ≤ 0.05 = *, 0.001 < *p* ≤ 0.01 = **, 0.0001 < *p* ≤ 0.001 = ***, *p* < 0.0001 = ****. a, b, c, d, and e stand for significant differences at the 0.05 level, the same as follows.

## 5. Conclusions

This study aimed to explore the role of the *ZmPR5* gene in maize resistance to *Fusarium verticillioides*. Through experiments such as gene cloning, bioinformatics analysis, subcellular localization, over-expression in *Arabidopsis thalian*a, yeast two-hybrid assays, phenotypic analysis of maize mutants, and expression pattern analysis, the following conclusions were drawn: The *ZmPR5* gene encodes 175 amino acids. It is an unstable hydrophobic protein containing a THN domain and a signal peptide. *ZmPR5* is localized in the nucleus and shows high homology with the rice PR-5 protein. When *ZmPR5* was over-expressed in *Arabidopsis thaliana* and inoculated with *Fusarium verticillioides*, the antioxidant enzyme activity of the plants was enhanced, the cell membrane stability was improved, and the chlorophyll content remained relatively stable. These results indicate that over-expressing this gene can enhance the resistance of *Arabidopsis thaliana* to the pathogen. In maize, after inoculation with the pathogen, the changes in relevant indicators of the *zmpr5ems* mutants showed that their resistance was lower than that of the wild-type B73. Trypan blue staining further confirmed that the cell membrane damage in the mutant was more severe.

Yeast two-hybrid assays showed that *ZmPR5* interacts with *WRKY53* and *glucA*, suggesting its important role in regulating the expression of disease resistance genes and the plant disease resistance process. In addition, treatments with ABA, MeJA, PEG6000, and NaCl could all induce the expression of *ZmPR5*, indicating that this gene has broad stress resistance functions.

In conclusion, *ZmPR5* plays a crucial role in plants’ defense against *Fusarium verticillioides* infection. It improves plants’ tolerance to the pathogen by enhancing antioxidant capacity, maintaining cell membrane stability, and participating in disease resistance-related regulation. This study provides a basis for a deeper understanding of the molecular mechanisms of plant–pathogen interactions and offers theoretical support for breeding new maize varieties resistant to ear rot.

## Data Availability

Data will be made available on request.
